# Effects of intelligence and approximate number system on the non-symbolic division ability in preschoolers

**DOI:** 10.3389/fpsyg.2023.961140

**Published:** 2023-06-23

**Authors:** Nayun Kwon, So-Yeon Kim

**Affiliations:** Department of Psychology, Duksung Women’s University, Seoul, Republic of Korea

**Keywords:** approximate number system, mathematics, division, intelligence, cognitive development, non-symbolic division ability

## Abstract

Recently, it has become evident that cognitive abilities such as the approximate number system (ANS), number knowledge, and intelligence affect individuals’ fundamental mathematical ability. However, it is unclear which of these cognitive abilities have the greatest impact on the non-symbolic division ability in preschoolers. Therefore, in the present study, we included 4- to 6-year-old Korean preschoolers without prior formal education of division in order to test their ability to solve non-symbolic division problems, ANS acuity, and intelligence, and to determine the interrelationships among those functions (*N* = 38). We used the Panamath Dot Comparison Paradigm to measure the ANS acuity, employed non-symbolic division tasks to measure the ability to solve non-symbolic division problems, and measured the intelligence using the Korean version of the WPPSI-IV (Wechsler Preschool Primary Scale of Intelligence-IV). Our results showed that, in all conditions of the non-symbolic division tasks, the 4- to 6-years old children were able to perform better than chance level. Additionally, in a relatively easy condition, the children’s performance showed a significant positive correlation with full-scale intelligence quotient (FSIQ) and ANS acuity; however, in a more complex condition, only FSIQ was significantly correlated with their performance. Overall, we found significant relationships between the children’s performance in the non-symbolic division tasks and verbal comprehension, fluid reasoning, and processing speed index. Taken together, our findings demonstrate that preschoolers without formal education on the arithmetic problem solving can solve non-symbolic division problems. Moreover, we suggest that both FSIQ and ANS ability play essential roles in children’s ability to solve non-symbolic division problems, highlighting the significance of intelligence on children’s fundamental mathematical ability.

## Introduction

Acquiring fundamental arithmetic skills is a crucial aspect of early education, and various cognitive abilities like approximate number system (ANS), number knowledge, and intelligence are linked to these skills. The relationship among these variables and early arithmetic skills, however, is yet to be fully specified (Mussolin et al., 2014; Elliott et al., 2019; Finke et al., 2020). Recent studies have provided evidence of a positive association between ANS ability and fundamental mathematical knowledge (He et al., 2016; Elliott et al., 2019; Finke et al., 2020).

ANS refers to the capacity to estimate the magnitude of a group without counting or relying on language or symbols (McCrink and Spelke, 2016; McCrink et al., 2017). Previous research has indicated that ANS is based on intrinsic intuition and innate cognitive abilities that are common in both adults and newborns, as well as animals (Dehaene, 1997; Feigenson et al., 2004). As ANS-related number identification relies on Weber’s ratio - which is based on the ratio between numbers rather than their absolute size – the identification process becomes faster and more accurate as the difference between two or more numbers increases (Xu and Spelke, 2000; Lipton and Spelke, 2003; Xu and Arriaga, 2007; Cho, 2013; Libertus et al., 2013). Previous studies have suggested that the ability to identify ratios develops gradually as children age. Additionally, using ANS, children can progress from making general comparisons of numbers to even performing approximate addition and subtraction (Halberda and Feigenson, 2008; Libertus et al., 2011; Kibbe and Feigenson, 2015).

There have been several recent studies on whether children who have not yet received formal mathematical education can use their ANS to solve non-symbolic arithmetic problems. Kibbe and Feigenson (2015) showed that children aged 4- to 6-year-old could not solve addition problems presented symbolically (using Arabic numerals or verbal number words), but the children were able to solve non-symbolic addition problems using piles of multi-colored buttons, pennies, or blue toy shoes. The task that Kibbe and Feigenson (2015) used did not explicitly require the ANS abilities, but the take was created in a way that promoted the use of ANS representations. Recent studies have investigated whether preschool children possess an innate ability to make approximate calculations without any formal training or education, and the results have shown that they can solve non-symbolic multiplication problems at a significantly higher rate than chance (Barth et al., 2009; McCrink and Spelke, 2010; Kwon et al., 2018). Furthermore, McCrink and Spelke (2016) demonstrated that 5- to 6-year-old children possess the capability to solve non-symbolic division problems by utilizing their ANS. Specifically, McCrink and Spelke (2016) indirectly examined the influence of ANS on non-symbolic division performance by utilizing division tasks that engaged the ANS. Although the researchers did not directly measure children’s ANS abilities, their research shed light on the effects of ANS on non-symbolic division tasks. Several longitudinal studies have also shown that non-symbolic processing is strongly related to later number knowledge and arithmetic abilities (Soto-Calvo et al., 2015; Geary et al., 2017; Szkudlarek and Brannon, 2017; Finke et al., 2020). Taken together, these results suggest that children have an innate understanding of multiplication and division that emerges independently of any formal education on symbolic multiplication and division.

However, Göbel et al. (2014) measured children’s ANS abilities and knowledge of Arabic numbers, which are known to be factors affecting the development of arithmetic abilities in early childhood. In their results, researchers reported that the ANS ability did not predict individuals’ future arithmetic abilities. Instead, the researchers demonstrated that six-year-olds’ knowledge of Arabic numbers was a strong longitudinal predictor for the arithmetic skill development (Göbel et al., 2014). Similarly, other studies failed to find significant correlations between ANS acuity and symbolic arithmetic abilities (Luculano et al., 2008; Holloway and Ansari, 2009; Obersteiner et al., 2013). Specifically, Geary et al. (2017) and Geary and vanMarle (2018) found that the ability to understand cardinality in preschoolers revealed a strong positive correlation with later arithmetic abilities and number system knowledge after controlling for intelligence, executive function, and parental education levels (Geary et al., 2017; Geary and vanMarle, 2018). Moreover, Finke et al. (2020) demonstrated that symbolic number processing showed a stronger association with later arithmetic ability than non-symbolic number processing (Finke et al., 2020). However, Finke et al. (2020) also reported that early non-symbolic processing significantly affected later symbolic number processing even after controlling for other cognitive factors. The researchers suggested that non-symbolic number processing ability facilitated symbolic number processing and promoted arithmetic skills (Finke et al., 2020).

Like ANS, intelligence can be also an important factor in mathematical learning and achievement, as evidenced by recent research (Deary et al., 2007). To better understand the relationship between intelligence and mathematical abilities, researchers have suggested breaking intelligence down into sub-factors such as working memory (WM), processing speed, visuospatial index, and verbal index (Chen and Li, 2014; Passolunghi et al., 2015). Passolunghi et al. (2015) examined the early mathematical abilities of children, including number comparisons, sequential comparisons, categorization, numeracy, numerical strength, and overall number knowledge. They also looked at intellectual abilities such as processing speed, WM, phonological and visuospatial short-term memory, and total intelligence. The results showed a significant correlation between processing speed and early mathematical abilities in 5- to 6-year-old children, as well as a significant correlation between early mathematical abilities and phonological ability. Verbal intelligence was found to have a direct impact on early mathematical abilities, while non-verbal intelligence was correlated with numerical abilities which was mediated by phonological abilities, processing speed, and WM. However, it is important to note that the numerical ability test used in Passolunghi et al. (2015) relied heavily on verbal abilities, and thus, the results may have been different if the test had been less reliant on verbal skills.

Overall, previous studies have yielded mixed results on the extent to which the ANS or intelligence influences the development of non-symbolic arithmetic abilities. Although some studies have found that intelligence plays a crucial role in children’s mathematical abilities, few have examined the interplay between early division ability, intelligence, and ANS abilities. Furthermore, previous research on the effects of ANS on children’s arithmetic abilities has not directly measured children’s ANS but has tested ANS effects on non-symbolic arithmetic performance through math tasks involving the ANS (McCrink and Spelke, 2010, 2016). Consequently, studies on investigating relationships among preschool children’s ANS abilities, intelligence, and non-symbolic division abilities are scarce (Barth et al., 2009). To fill this research gap, we used the Panamath program to measure children’s ANS abilities without involving arithmetic abilities and examined the interrelationships among non-symbolic division ability, ANS, and intelligence. In addition to such manipulation, we expanded previous research findings on non-symbolic division abilities to preschoolers. In detail, previous studies mainly recruited 5- to 7-year old (McCrink and Spelke, 2010) or 5- to 6-years old children (McCrink and Spelke, 2016) to determine whether young children who had not received prior education on division or multiplication could solve non-symbolic arithmetic problems. Importantly, we expanded previous research on non-symbolic division abilities to preschoolers aged 4–6 years old to explore whether children who have not received formal elementary education could solve non-symbolic division problems. Our choice of preschoolers as participants is based on the fact that sustained attention and schematic of non-symbolic numbers develop rapidly in children aged 2–4 years old (Levy, 1980; Ruff and Rothbart, 2001). Also, previous literature has demonstrated that children as young as 4 years old can complete lengthy tasks (Resnick, 1989).

## Present study

The aim of the current study is to investigate the relationships between the ANS, intelligence, and the ability to solve non-symbolic division problems in preschool-aged children (aged 4–6) with no prior education in division. Specifically, we hypothesized that: (1) preschool children (aged 4-to-6) who have not received prior education on solving arithmetic problems can solve non-symbolic division problems, (2) ANS ability will be significantly correlated with non-symbolic division ability in preschoolers, (3) the ratio between the number sets to be compared (1:1.5 and 1:2.0, respectively; described below) will influence the children’s responses in non-symbolic division tasks, (4) intelligence will be significantly correlated with preschooler’s non-symbolic division ability, and lastly, (5) we aimed to investigate how the ANS ability, intelligence, and non-symbolic division ability were related to each other by examining them in combination and identifying the key factors influencing the non-symbolic division ability in preschool children.

## Materials and methods

### Participants and procedure

#### Participants

Thirty-eight preschoolers (22 boys and 16 girls, age range: 48–80 months, average age: 63 months) participated in the current study. There were fifteen 4-years old children, eleven 5-years old children, and twelve 6 years-old children in our participants. None of the participants had prior education of division or multiplication and all of them have normal visions (20/20 vision). All participants were recruited through Internet advertisements. Written informed consents from all participants’ parents or guardians were obtained prior to participation. A week after participating in our study, all children and their parents were provided with the child’s full-scale IQ scores, an interpretation of the IQ scores, and scores on the ANS task as compensation. Also, all children were provided with small sweets (a candy box) right after the study under the parent’s agreement. All protocols and procedures of the study were approved by the institutional review board of the University (IBR No. 2016-011-006).

#### Procedures

All children performed the following three experimental tasks: (1) an ANS task, (2) a non-symbolic division task, and (3) the Korean version of the Wechsler Infant Intelligence Test-IV (K-WPPSI-IV). The order of the tasks was counterbalanced among participants. All tasks were conducted in an 1:1 format comprising each child and a researcher in an independent laboratory. The ANS task lasted for about 3 min and the average time for the non-symbolic division tasks and the intelligence task was 30 min and 1 h and 10 min, respectively. Overall, the total procedure took 2 h on average. All participants were provided with their K-WPPSI-IV and ANS measurement results after a week from the experiment.

### Materials and methods

#### Panamath task: testing ANS

**Figure 1:**
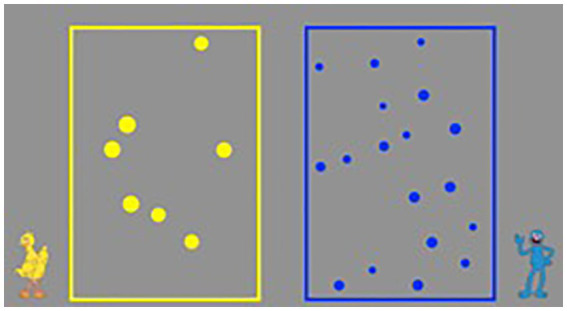
Panamath ANS task (Halberda et al., 2008). Permissions and licenses from the rights holders (Dr. McCrink & Dr. Halberda).

We used the Panamath program,^1^ developed by Halberda et al. (2008), to measure the participants’ ANS abilities. The total trials of the ANS were 70 trials. Each trial lasted until participant’s response. The Panamath measures’ test–retest reliability of the Weber fraction was reported as *r* = 0.78 (Halberda et al., 2012) and split-half reliability of mean accuracy was reported as *r* = 0.69 (Libertus et al., 2016) in the previous literatures. As shown in Figure 1, Panamath presented non-symbolic discrimination tasks in which an array of yellow dots and an array of blue dots were presented simultaneously on opposite sides of the screen. Participants were asked to quickly answer which color array contained more dots. To minimize experimenter effects, the experimenter sat behind the child, maintaining his/her gaze on the child, until the child responded, and looked at the computer screen after the child had provided his/her response. Each stimulus was presented for approximately 3.23 s to prevent children from counting objects (Halberda et al., 2008). When a participant responded to a stimulus, a screen with a mixture of yellow, blue, and white dots was briefly presented, followed by a screen with a fixation point on a gray background. When the participant was ready to perform the next trial, the experimenter pressed the space bar on the keyboard, then the next stimulus appeared. The difficulty level of each trial was randomly chosen among the ratios of 1.2:1, 1.3:1, 1.8:1, and 3:1 (e.g., the 1.3:1 ratio may present 1.3 yellow dots for each blue dot).

#### Non-symbolic division task

To examine the children’s ability to solve non-symbolic division problems, we adapted the procedures and task paradigm used in McCrink and Spelke (2010, 2016). The division experiment comprised three blocks: intro, training, and test. There were two task conditions for division: divide-by-two (Division 2) and divide-by-four (Division 4); these conditions were presented in three blocks each, making a total of six blocks in the task.

**Intro Block.** The purpose of the intro block was to help participants understand the division task. For example, in the Division 2 condition, participants were initially presented with two blue rectangles that changed in size (i.e., increasing or decreasing) over the course of several seconds. Then, the rectangles stopped changing size and a magic wand appeared on the left side of the screen, sweeping up and down the rectangles with a shimmering magical sound. After several seconds, the two rectangles merged, and the experimenter said: “Look! Our magic dividing wand! Originally, there were two blue rectangles, and now there is one rectangle. It reduced the quantity. The magic wand made the two blue rectangles become one!” After watching this short clip, the participant watched another transformation trial movie. This transformation movie was identical to the previous one, but when the magic wand appeared, the rectangles were obscured by a screen. After the wand had finished waving, the video was paused and the participant was asked to try to guess the number of rectangles behind the screen. If the child gave the correct answer, he/she could advance to the training block. In our study, only one child provided an incorrect answer on the first attempt (he/she then answered correctly on his/her second attempt); all of the other children were able to respond correctly on their first attempt.

**Figure 2:**
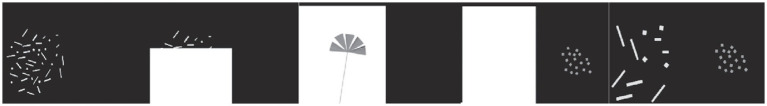
Non symbolic division task – Training block (McCrink and Spelke, 2010). Reprinted from McCrink and Spelke (2010) with permission from Elsevier.

**Training Block.** In the training block, an array of blue rectangles was presented on the left side of the screen. The experimenter pointed to the array and said: “Now, there are many rectangles. There are too many rectangles to count. We will concentrate and use our imagination.” After 5 seconds, the array was obscured, and the magic division wand appeared above it. The experimenter said, “Look! The rectangles are dividing,” At this time, a comparison array comprising pink rectangles appeared on the right side of the screen. When the movie was paused, children were asked to choose the side (left or right) that contained more rectangles (Figure 2). The experimenter sat behind the child, asked neutral questions (e.g., “Which side of the screen do you think has more rectangles?”), and let the participant answer. When the child responded, the experimenter recorded the response and re-played the trial movie. When the video was played back, the obscuring screen was removed, providing feedback. The training block comprised six trials.

As in McCrink and Spelke (2016), we manipulated a *distance* factor in the division task. We set this factor by setting the relationship (or “distance”) between the comparison array and the transformed array (i.e., the correct outcome) to be relatively disparate or close. For the disparate conditions, the number of rectangles in the two arrays differed by a factor of 2.0, while for the close conditions, they differed by a factor of 1.5. There were four distance conditions, /2.0, *2.0, /1.5, and *1.5, respectively, and these were applied in both division conditions. For example, in the disparate conditions (i.e., /2.0, *2.0), the number of small rectangles in the comparison array was either double (*2) or half (/2) that of the transformed array. In the close conditions (i.e., /1.5, *1.5), the value of the comparison array was either the correct amount *1.5 or the correct amount /1.5. For example, if an initial array comprised 16 rectangles, the correct outcome in the Division 2 condition would be eight rectangles. If the disparate condition was applied to this example, the number of rectangles in the comparison array would be four or 16, while the number would be six or 12 when the close condition was applied. That is, trials featuring the disparate condition were easier than those featuring the close condition, as arrays with a distance factor of 2.0 were more discriminable than arrays with a distance factor of 1.5. As the previous study has already shown the effects of the distance conditions, we used the same manipulation for the distance. For more details, please refer to McCrink and Spelke (2016).

**Figure 3:**
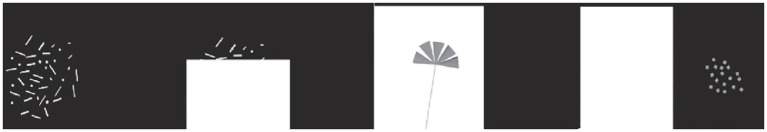
Non symbolic division task – Test block (McCrink and Spelke, 2010). Reprinted from McCrink and Spelke (2010) with permission from Elsevier.

**Test Block.** The 16 test trials were conducted identically to the training block, with the following three exceptions: (1) neither correct answers nor feedback were provided for children’s responses. Instead, the experimenter provided consistent positive feedback (e.g., “Good job!” and “Let us try another one!”), (2) in the training trials, the stimuli in the comparison array were all small rectangles of unified size; however, in the test block, the stimuli in the comparison arrays were rectangles and, for all distance conditions, each array occupied the same area and had the same contour length (Figure 3). Thus, if a child made numerical comparisons simply based on the area and/or length of the array, he/she would perform below chance level in the test block. For a child to successfully solve a problem, he/she needed to consider the exact change in the number of rectangles, not the perceptual variables that changed depending on the value of the array, and (3) if participants seemed to count the numbers using their fingers or by moving their lips, the experimenter intervened in the test block. Each trial lasted until the participant made their choice.

#### Intelligence test

We used the K-WPPSI-IV to examine children’s intelligence and sub-scale scores (Lee et al., 2016). Unlike previous K-WPPSI versions, which can only examine fluid, crystallized, and total intelligence, we chose the latest version to examine verbal comprehension, visuospatial, fluid reasoning, and processing speed abilities (Lee et al., 2016). Of the 15 subtests contained in the K-WPPSI-IV, 10 subtests, comprising six basic subtests and four supplementary subtests, were used to measure the participants’ total intelligence and five sub-indexes of intelligence (verbal comprehension index, visuospatial index, fluid reasoning index, WM index, and processing speed index, respectively). Since two children were tested their intelligence within the last 3 months (8 and 10 weeks in advance), additional intelligence tests were not conducted in this study due to the reliability issue. Thus, one child provided only total intelligence score and the other child tested different version of intelligence test (K-WISC-III) and provided only four sub-indexes. We included the data from the child in our analyses because all different version of Wechsler scale of Intelligence for children are resemble each other as they have been derived out of the same original Wechsler scale. Moreover, since we included only FSIQ scores for the further analysis (except for the correlation analysis), we decided not to exclude these two participants. Table 1 shows the mean scores and standard deviations for each of the five sub-indexes.

**Table 1:** Descriptive statistics of K-WIPPSI-IV intelligence test.

	*N*	Min.	Max	Average	SD
Full scale IQ	38	77	133	108.63	13.37
Verbal comprehension (VCI)	37	74	127	109.89	12.76
Visuospatial (VSI)	36	73	152	107.03	15.16
Fluid reasoning (FRI)	37	63	128	107.76	15.11
Working memory (WMI)	37	67	132	106.59	15.09
Processing speed (PSI)	37	67	131	102.32	14.06

## Results

### Non-symbolic division task

As shown in Table 2, the overall performance on both division tasks was significantly higher than the chance level. Specifically, for the Division 2 condition, the accuracy rate was 69.24% in the training block and 72.04% in the test block (*t*s (37) = 7.72 and 13.13, respectively; *p*s < 0.001), while for the Division 4 condition, the children showed an 80.80% accuracy rate for the training block and a 69.74% rate in the test block (*t*s (37) = 12.50 and 9.45, respectively; *p*s < 0.001).

**Table 2:** Descriptive statistics of division task accuracy in test block.

	*N*	Average (%)	SD	*t*	*p*
Division 2	38	72.04%***	10.35	13.13	<0.001
Division 4	38	69.74%***	12.88	9.45	<0.001

To test whether the effects of distance condition differed for each age group, a 4 (distance; within-subjects factor) × 2 (division difficulty; within-subjects factor) × 3 (age; between-subjects factor) mixed analysis of variance (ANOVA) was conducted. The results revealed a significant main effect of distance (*F*(1,35) = 15.53, *p* < 0.001, *η_p_*^2^ = 0.307) and difficulty (*F*(1,35) = 8.48, *p* < 0.01, *η_p_*^2^ = 0.195), but the main effect of age was not significant (*F*(1,35) = 2.508, *p* = 0.096, *η_p_*^2^ = 0.125). Also, the interactions between distance and age (*F*(2,35) = 2.303, *p =* 0.115, *η_p_*^2^ = 0.116) and the interaction between the difficulty and age (*F*(2,35) = 0.265, *p* = 0.769, *η_p_*^2^ = 0.015) were not significant. Importantly, however, the interaction between distance and difficulty (*F*(1,35) = 4.5, *p* < 0.05, *η_p_*^2^ = 0.114) and a three way interaction among the factors was significant (*F*(2,35) = 4.34, *p* < 0.05, *η_p_*^2^ = 0.199).

**Figure 4:**
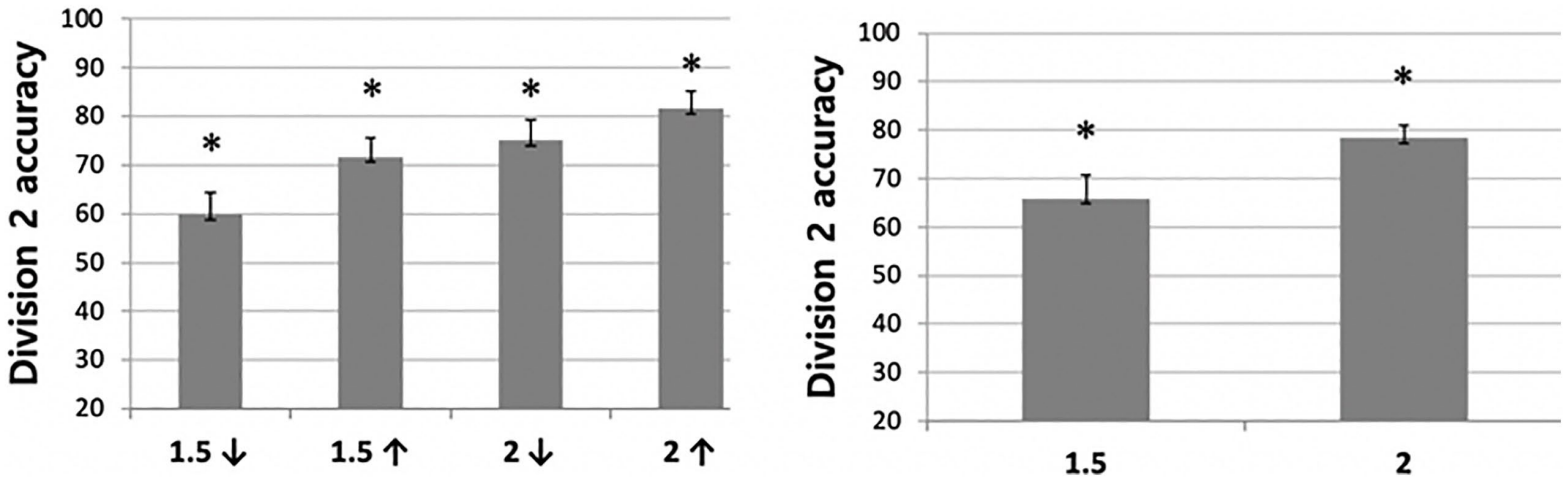
Division 2 accuracy difference between distance conditions. The error bars of figures display standard errors.

To better understand the significant three-way interaction in the first analysis, we conducted additional 4 (distance; within-subjects factor) × 3 (age; between-subjects factor) mixed analyses of variance (ANOVA) for each division condition (i.e., easy and difficult ones). The results from the ANOVA on the accuracy data in the Division 2 condition revealed a significant main effect of distance (*F*(1,35) = 18.56, *p* < 0.001, *η_p_*^2^ = 0.347), but the main effect of age (*F*(1,35) = 0.900, *p* = 0.416, *η_p_*^2^ = 0.049) and an interaction between the two factors were not significant (*F*(2,35) = 0.609, *p* = 0.550, *η_p_*^2^ = 0.034). As shown in Figure 4, participants performed significantly better than chance level in all distance conditions. Additionally, all children performed significantly better in the disparate than the close condition. Follow-up pairwise comparisons showed significantly lower performance in the /1.5 condition (59.87%) than other conditions. That is, accuracy in the /1.5 condition was significantly lower than *1/5 condition (71.71%, *t* (37) = −2.018, *p* = 0.051), /2.0 condition (75%, *t*(37) = −3.38, *p* < 0.01) and *2.0 condition (81.58%, *t*(37) = −3.883, *p* < 0.001). Moreover, children’s performance was above chance level for all distance conditions (for /1.5, *1.5, /2. *2; *t*s (37) = 2.43, 6.35, 6.43, and 9.80, respectively; all *p*s < 0.01).

**Figure 5:**
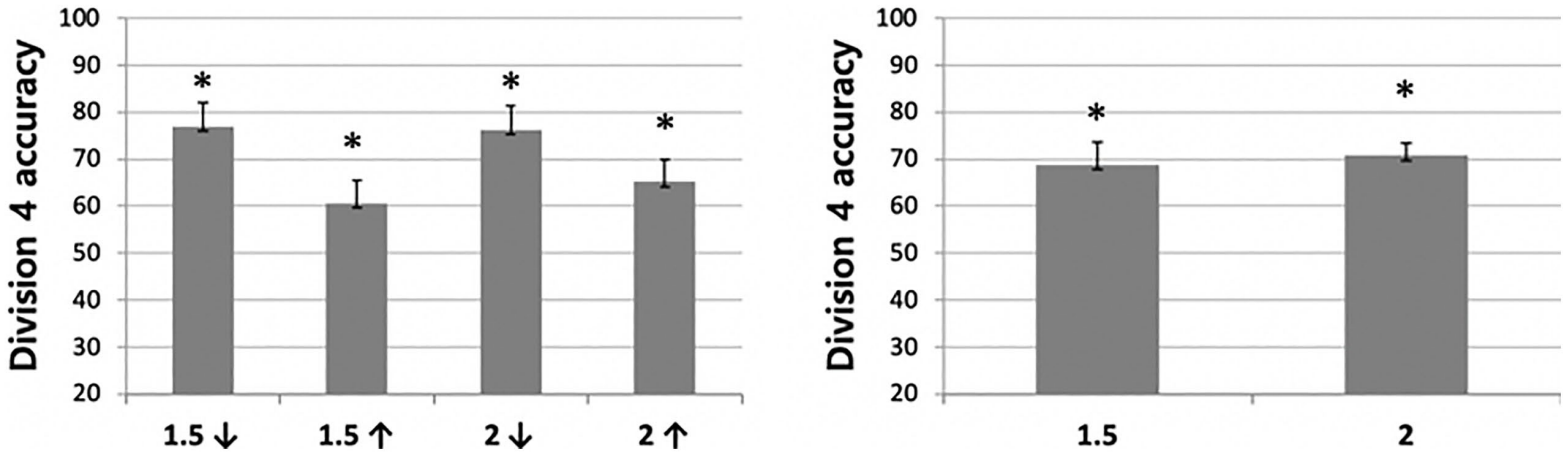
Division 4 accuracy difference between distance conditions.

The same ANOVA was conducted for the Division 4 condition. Unlike the results for the Division 2 condition, main effects of age (*F*(1,35) = 3.116, *p* = 0.057, *η_p_*^2^ = 0.151) and distance (*F*(1,35) = 0.582, *p* = 0.450, *η_p_*^2^ = 0.016) were not significant. Moreover, neither the main effect of age (*F*(2,35) = 3.116, *p* = 0.057, *η_p_*^2^ = 0.151) nor the interaction between the two factors were significant (*F*(2,35) = 0.979, *p* = 0.443, *η_p_*^2^ = 0.053). The children performed significantly better than chance level in all distance conditions (Figure 5); however, differing from the findings for the Division 2 condition, there was no significant difference between the close and disparate distance conditions. That is, all participants, including those as young as 4 years of age, performed significantly well in the Division 4, regardless of the distance condition.

### Approximate number system

**Figure 6:**
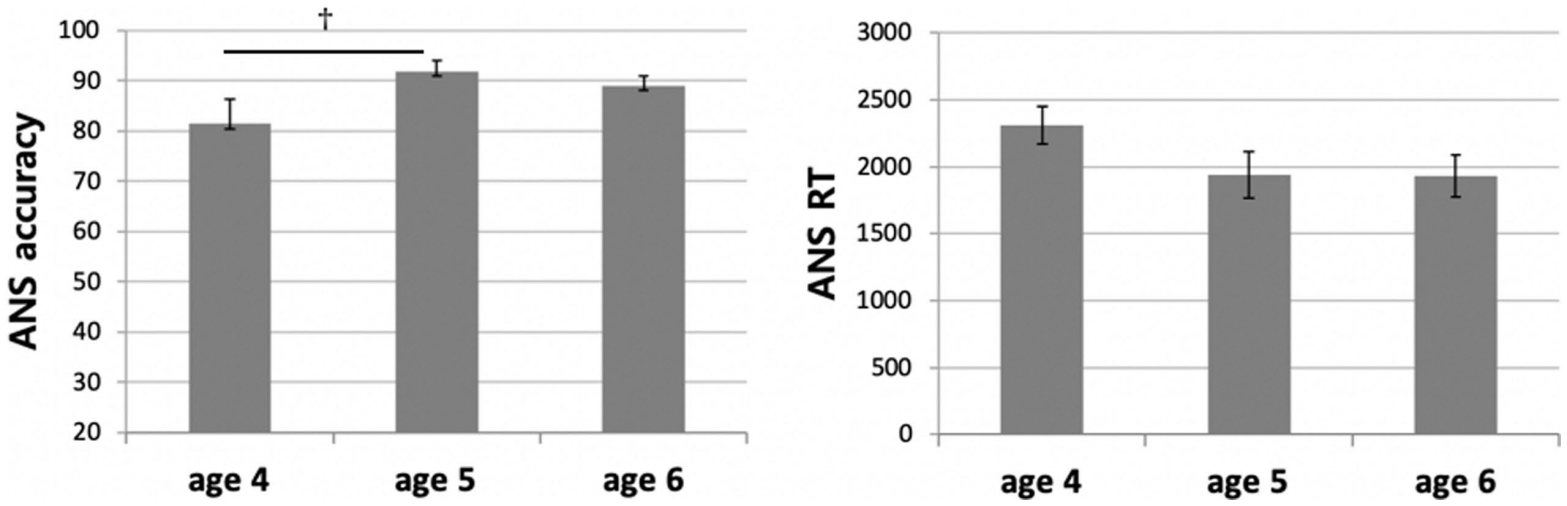
ANS accuracy differences (left) and ANS RT differences (right) between age groups.

We conducted two one-way ANOVAs on accuracy and reaction time (RT) data, with age as a between-subjects factor. The results revealed a significant main effect of age on the ANS accuracy (*F*(2,35) = 3.441, *p* < 0.05, *η_p_*^2^ = 0.164; Figure 6), but the effect was insignificant on RT (*F*(2,35) = 0.899, *p* = 0.416, *η_p_*^2^ = 0.049; Figure 6). The *post-hoc* multiple comparisons (Bonferroni-corrected) revealed a marginally significant difference between the four- and five-year-old children on ANS accuracy (95% Confidence Interval [−21.06, 0.14], *p* = 0.054), but the difference between the four- and six-year-old group was not significant (95% CI [−17.92, 2.76], *p* = 0.221). There was no significant difference in this regard between the five- and six-year-olds (95% CI [−8.26, 14.02], *p* = 1.000).

### Relationships among performance in division tasks, ANS, and intelligence

To examine the relationship among performance in the division tasks, ANS accuracy, and intelligence, Pearson correlation analysis was performed. As shown in Table 3, factors that had a significant correlation with the children’s Division 2 accuracy were found to be Division 4 accuracy (*r* = 0.32, *p* < 0.05), ANS accuracy (*r* = 0.36, *p* < 0.05), full-scale IQ (FSIQ; *r* = 0.52, *p* < 0.01), fluid reasoning index (FR index; *r* = 0.48, *p* < 0.01), and processing speed index (*r* = 0.40, *p* < 0.05). Factors that showed a significant correlation with the Division 4 accuracy were age (*r* = 0.32, *p* < 0.05), Division 2 accuracy (*r* = 0.39, *p* < 0.05), FSIQ (*r* = 0.45, *p* < 0.01), verbal comprehension index (*r* = 0.42, *p* < 0.05), FR index (*r* = 0.36, *p* < 0.05), Working memory index (*r* = 0.36, *p* < 0.05), and processing speed index (*r* = 0.41, *p* < 0.05). Additionally, ANS accuracy was positively correlated with age (*r* = 0.35, *p* < 0.05) and processing speed index (*r* = 0.40, *p* < 0.05).

**Table 3:** Correlate relationship between division, ANS, and intelligence.

		1	2	3	4	5	6	7	8	9	10	11
1	Age	1										
2	Division 2	0.12	1									
3	Division 4	0.32*	0.39*	1								
4	ANS Accuracy	0.35*	0.36*	0.12	1							
5	ANS RT	−0.29	−0.11	−0.19	0.16	1						
6	FSIQ	0.14	0.52**	0.45**	0.29	−0.17	1					
7	VCI	0.15	0.29	0.42*	0.15	0.02	0.68**	1				
8	VSI	−0.07	0.33	0.16	0.22	0.02	0.58**	0.21	1			
9	FRI	0.09	0.48**	0.36*	0.09	−0.10	0.62**	0.36*	0.27	1		
10	WMI	0.19	0.14	0.36*	0.20	−0.19	0.63**	0.18	0.34*	0.25	1	
11	PSI	0.19	0.40*	0.41*	0.40*	−0.18	0.61**	0.28	0.48**	0.29	0.39*	1

To examine the effects of FSIQ and ANS accuracy on the non-symbolic division ability, a hierarchical regression analysis was conducted. Division accuracy was set as a dependent variable and age, ANS accuracy, and children’s FSIQ were set as independent variables. Specifically, for Model 1, we set the age as a predicting variable to identify the effect of age on division abilities. For Model 2, we included ANS accuracy as an additional variable. In Model 3, we further introduced FSIQ as an independent variable to explore the effects of both ANS accuracy and FSIQ on preschoolers’ non-symbolic division ability while controlling for age. The results indicated that the influence of age on Division 2 task accuracy was not significant (*R*^2^ = 0.001, *β* = 0.030, *p* = 0.857). Notably, as presented in Table 4, the addition of ANS accuracy in the second model significantly enhanced the explanatory power to 13.4% (*R*^2^ = 0.134, *⊿R*^2^ = 0.084, *p* < 0.05). Additionally, FSIQ of the children significantly predicted Division 2 task accuracy (FSIQ: *β* = 0.454, *p* < 0.01) in the third model, and this model accounted for 32.2% of the total variance in Division 2 accuracy.

**Table 4:** Hierarchical regression analysis of Division 2, ANS, and intelligence after controlling for age.

Dependent variable	Model	Independent variable	*B*	SE	*β*	t(*p*)	VIF
Division 2	Model 1	Age	0.369	2.027	0.030	0.182	1.000
		*F(p) = 0.*033, *R*^2^ = 0.001, adj.*R*^2^ = −0.027					
	Model 2	Age	−1.034	2.008	−0.085	−0.515	1.100
		ANS correction	0.350	0.151	0.382	2.316*	1.100
		*F(p) =* 2.700*, *R*^2^ = 0.134, adj.*R*^2^ = 0.084					
	Model 3	Age	−1.200	1.804	−0.099	−0.665	1.101
		ANS correction	0.232	0.141	0.253	1.640	1.189
		FSIQ	0.351	0.114	0.454	3.068**	1.096
		*F(p)* = 5.371**, *R*^2^ = 0.322, adj.*R*^2^ = 0.262					

For the Division 4 task, the effect of age on the accuracy was not significant (*R*^2^ = 0.090, *β* = 0.300, *p* = 0.067). As shown in Table 5, in the results of the second model where ANS accuracy was added, the explanatory power insignificantly increased to 9.1% (*R*^2^ = 0.091, *⊿R*^2^ = 0.039, *p* = 0.838). Lastly, children’s FSIQ was added as independent variable for Model 3 to examine their effects on division tasks after controlling for age. FSIQ significantly predicted the Division 4 accuracy (*β* = 0.449, *p* < 0.01) and the explanatory power significantly increased to 27.5% (*R*^2^ = 0.275, *⊿R*^2^ = 0.211, *p* < 0.01) after controlling for the effects of age. However, ANS accuracy were not significant factors explaining the variances in Division 4 accuracy. Taken together, our findings indicate that in Model 2, ANS ability had a significant positive impact on Division 2 accuracy. However, when intelligence was introduced in Model 3, the influence of ANS ability became statistically insignificant. Moreover, only FSIQ demonstrated a significant association with Division 4 accuracy. Consequently, our results suggest that intelligence plays a pivotal role in children’s overall accuracy when solving non-symbolic division problems.

**Table 5:** Hierarchical regression analysis of Division 4, ANS, and intelligence after controlling for age.

Dependent variable	Model	Independent variable	*B*	SE	*β*	t(*p*)	VIF
Division 4	Model 1	Age	4.548	2.407	0.300	1.889	1.000
		*F(p)* = 3.570, *R*^2^ = 0.090, adj.*R*^2^ = 0.065					
	Model 2	Age	4.389	2.559	0.290	1.715	1.100
		ANS correction	0.040	0.193	0.035	0.206	1.100
		*F(p)* = 1.759, *R*^2^ = 0.091, adj.*R*^2^ = 0.039					
	Model 3	Age	4.184	2.320	0.276	1.804	1.101
		ANS correction	−0.106	0.182	−0.093	−0.585	1.189
		FSIQ	0.432	0.147	0.449	2.936*	1.096
		*F(p) =* 4.300*, *R*^2^ = 0.275, adj.*R*^2^ = 0.211					

## Discussion

In the present study, we directly compared the effects of the ANS and intelligence on preschoolers’ performance of non-symbolic division. Specifically, we tested preschoolers regarding their ability to solve non-symbolic division. Previous studies have shown that children as young as 5- to 7-years without prior exposure to arithmetic problems can solve non-symbolic division problems (McCrink and Spelke, 2016); however, whether preschool children aged 4- to 6-years could also solve such problems remained unknown. In the present research, we reported for the first time that preschoolers aged 4- to 6-years can also solve non-symbolic division problems above the chance level.

Previous studies testing the influence of ANS on children’s arithmetic abilities did not directly measure the ANS (McCrink and Spelke, 2010, 2016). Specifically, McCrink and colleagues tested the effects of ANS on division-reasoning performance by setting division tasks that contained conditions which activated the ANS. However, it was not clarified in such previous studies whether young children actually had strong ANS abilities. Moreover, the effects of age on ANS performance were not directly tested in the previous studies. In contrast, here, we directly measured the children’s ANS abilities using the Panamath program, separating this ability from the non-symbolic division task. Thus, we were able to test the direct relationship between ANS and non-symbolic division in preschool children. Furthermore, we conducted FSIQ tests and directly examined the relationships among sub-components of the FSIQ, children’s ANS ability, and division-reasoning ability.

Our findings revealed that preschoolers performed above chance level in both the Division 2 and Division 4 non-symbolic division tasks. These findings suggest that preschoolers aged 4- to 6-years old, without formal education in division, are capable of solving non-symbolic division-reasoning problems. Consistent with expectations, children exhibited significantly higher performance in the 2.0 distance condition (i.e., when the difference between the value of the comparison array and the obscured array varied by a factor of 2.0; either /2.0 or *2.0) compared to the 1.5 distance condition, for both the Division 2 and Division 4 conditions.

For Division 2 accuracy, ANS accuracy revealed a significant effect, but when intelligence was added as an additional factor, the effect of ANS became insignificant. That is, we found that FSIQ was determined to be the strongest predictor for non-symbolic division ability, after controlling for the effects of age and the ANS ability. In other words, intelligence is more crucial factor than age or ANS ability in solving non-symbolic division problems. These results are consistent with our recent findings for the non-symbolic multiplication performance in preschoolers (Kwon et al., 2018). In the previous study, we found ANS played a significant role in the multiplication 2 task after controlling for effects of FSIQ, but not in the multiplication 4 task. Consequently, we proposed that the multiplication 4 condition required more WM capacity and effort to manipulate the larger number of transformations. Consistent with our earlier research (Kwon et al., 2018), in the current study, we found a significant correlation between WM and Division 4 task accuracy, but not in the Division 2 task. It can be interpreted as that, in order to perform these tasks, children aged 4- to 6-years old must have remembered two or more approximate quantities and then manipulated or combined them under the Division 4 condition which were more difficult than that of the Division 2 condition. Prior studies have also identified a relationship between WM and the ANS and later arithmetic ability, and have shown that WM of preschool-aged children not only played an important role in later arithmetic abilities, but also in their ANS abilities (Kibbe and Feigenson, 2015; Passolunghi et al., 2015).

However, other studies have suggested that the ANS, an innate ability that cannot be explained by general cognitive abilities such as intelligence or attention, is especially related to mathematical achievement. One of these prior studies reported that children with developmental dyscalculia showed impaired brain mechanisms in regard to the ANS, while other areas, such as the language domain, showed no impairment (Kucian et al., 2006). Furthermore, 10-year-old children with developmental dyscalculia showed Weber’s ratio that was similar to five-year-old typically developing children; this might be due to slower development of the ANS in those children with developmental dyscalculia (Piazza et al., 2010; Mazzocco et al., 2011). Taken together with such previous findings, we suggest that both intelligence and ANS ability are essential factors in the development of mathematical abilities.

In the current study, some limitations are worth to be addressed. First, the number of participants in the current study was relatively small. This means that the results would have been sensitive to a case of several children producing extremely low values or more prone to extreme values so that make it difficult to generalize the result. However, the number of participants in previous studies testing non-symbolic arithmetic ability in children were even smaller than the present one. Moreover, a Shapiro-Wilk test was performed to examine if our division data followed a normal distribution to ensure our tests. The result did not show evidence of non-normality (Division 2: Ws < 0.9, *p*-values >0.5; Division 4: Ws < 1, *p*-values >0.05). Additionally, we used skewness and kurtosis of the distribution to assess normality, and the skewness of Division 2 and Division 4 accuracy were found to be under 1 and the kurtosis of Division 2 and Division 4 accuracy were also found to be under 1. Hair et al. (2010) and Byrne (2010) argued that data are considered to be acceptable in order to prove normal distribution if skewness is between −2 to +2 and kurtosis is between −7 to +7. Therefore, it is hard to conclude that the result of this study is due to extreme values. More importantly, a priori power analysis was conducted using G*Power version 3.1 (Faul et al., 2007) to determine the minimum sample size required to test the study hypothesis. For the ANOVA repeated measure analysis, the required sample size to achieve 80% power for detecting a medium effect (*f* ≥ 0.25) at a significance criterion of α = 0.05, was a total *N* = 36. Nonetheless, it will be valuable to replicate our results with different sets of participants in the future study to generalize the findings.

The second limitation could be that our division task only involved dividing by two or four conditions, meaning all correct answers were either half or quarter of the original. Similar to McCrink and Spelke’s (2016) study, we found that children’s performance was lower in the Division 4 condition than Division 2 condition. It is difficult to determine whether this lower performance is due to the differences in the initial amount or the size of the dividing number, which might be due to the scaling factor. However, based on the results in McCrink and Spelke (2010, 2016), the initial amount of the Division 2 condition was four times larger than the multiplication 2 condition, but the children showed similar performance in both conditions. Moreover, we calculated an ICC estimate [ICC (2,1)] to test measurement agreement for the division tasks. The result showed that the ICC value was 0.548 indicating the level of reliability can be regarded as acceptable (ICCs = 0.548 (95% CI, 0.135 ~ 0.764), *p* = 0.009).

Regarding the Division 4 condition, it can be possible that the lower performance of the Division 4 condition may due to the changes in the size of scaling factor itself. To clarify this, testing other variables which affect non-symbolic number processing can be helpful. Such variables can be indexes of spatial processing, numerical proportional reasoning, or symbolic number estimation (Boyer et al., 2008; Barth et al., 2009; McCrink and Spelke, 2010, 2016). However, in the current study, we did not directly examine above mentioned variables. Hence, it is helpful to examine effects of those continuous variables on division performance in future studies.

Despite the above mentioned limitations, to our knowledge, our study demonstrates for the first time that preschoolers aged 4- to 6-years can solve non-symbolic division problems at a significantly higher performance rate than chance level. These results extend existing findings that young children aged 5- to 7-years can solve non-symbolic arithmetic problems presented in non-symbolic form (Barth et al., 2009; McCrink and Spelke, 2010, 2016; Kibbe and Feigenson, 2015). Furthermore, we independently tested children’s ANS ability and division ability, and demonstrated significant effects of ANS and intelligence for non-symbolic division abilities in preschool children. Our findings suggest that even very young children who have no prior numerical or arithmetic knowledge can solve complex arithmetic problems by using their ANS. Consequently, we suggest that presenting arithmetic-reasoning problems in a non-symbolic way or training young children to approximately compare different quantities may play a crucial role in promoting children’s ability to reason unknown quantities and arithmetic problems. Future studies employing longitudinal designs and other cognitive factors (e.g., number knowledge, the ability to count, etc.) may expand our findings of effects of ANS and intelligence on non-symbolic arithmetic abilities to mathematic performance in school age children.

## Data availability statement

The datasets presented in this article are not readily available because due to the sensitive nature of the questions asked in this study, survey respondents were assured raw data would remain confidential and would not be shared. Requests to access the datasets should be directed to S-YK, vicky47syk@duksung.ac.kr.

## Ethics statement

The studies involving human participants were reviewed and approved by Institutional Review Board (IRB) of Duksung Women’s University. Written informed consent to participate in this study was provided by the participants’ legal guardian/next of kin.

## Author contributions

NK: conceptualization, data curation, writing-original draft preparation, and methodology. S-YK: conceptualization, supervision, validation, draft-reviewing and editing, and funding acquisition. All authors contributed to the article and approved the submitted version.

## Funding

This work was supported by the Ministry of Education of the Republic of Korea and the National Research Foundation of Korea (NRF-2020S1A5A8044530).

## Conflict of interest

The authors declare that the research was conducted in the absence of any commercial or financial relationships that could be construed as a potential conflict of interest.

## Publisher’s note

All claims expressed in this article are solely those of the authors and do not necessarily represent those of their affiliated organizations, or those of the publisher, the editors and the reviewers. Any product that may be evaluated in this article, or claim that may be made by its manufacturer, is not guaranteed or endorsed by the publisher.

## Footnotes

^1^ http://panamath.org

## References

[ref1] Barth H. Baron A. Spelke E. Carey S. (2009). Children’s multiplicative transformations of discrete and continuous quantities. J. Exp. Child Psychol. 103, 441–454. doi: 10.1016/j.jecp.2009.01.014, PMID: 19289237

[ref2] Boyer T. W. Levine S. C. Huttenlocher J. (2008). Development of proportional reasoning: where young children go wrong. Dev. Psychol. 44, 1478–1490. doi: 10.1037/a0013110, PMID: 18793078 PMC2597581

[ref3] Byrne B. M. (2010). Structural equation modeling with AMOS: Basic concepts, applications, and programming. New York: Routledge.

[ref4] Chen Q. Li J. (2014). Association between individual differences in non-symbolic number acuity and math performance: a meta-analysis. Acta Psychol. 148, 163–172. doi: 10.1016/j.actpsy.2014.01.016, PMID: 24583622

[ref5] Cho S. (2013). A review of the neurocognitive mechanisms of number sense. Korean J. Cogn. Sci. 24, 271–300. doi: 10.19066/cogsci.2013.24.3.003

[ref6] Deary I. J. Strand S. Smith P. Fernandes C. (2007). Intelligence and educational achievement. Intelligence 35, 13–21. doi: 10.1016/j.intell.2006.02.001

[ref7] Dehaene S. (1997). The number sense. Oxford University Press, Penguin press, NewYork, Cambridge (UK).

[ref8] Elliott L. Feigenson L. Halberda J. Libertus M. E. (2019). Bidirectional, longitudinal associations between math ability and approximate number system precision in childhood. J. Cogn. Dev. 20, 56–74. doi: 10.1080/15248372.2018.1551218

[ref9] Faul F. Erdfelder E. Lang A.-G. Buchner A. (2007). G*power 3: a flexible statistical power analysis program for the social, behavioral, and biomedical sciences. Behav. Res. Methods 39, 175–191. doi: 10.3758/BF03193146, PMID: 17695343

[ref10] Feigenson L. Dehaene S. Spelke E. (2004). Core systems of number. Trends Cogn. Sci. 8, 307–314. doi: 10.1016/j.tics.2004.05.002 15242690

[ref11] Finke S. Freudenthaler H. H. Landerl K. (2020). Symbolic processing mediates the relation between non-symbolic processing and later arithmetic performance. Front. Psychol. 11:549. doi: 10.3389/fpsyg.2020.00549, PMID: 32273864 PMC7113405

[ref12] Geary D. C. vanMarle K. (2018). Growth of symbolic number knowledge accelerates after children understand cardinality. Cognition 177, 69–78. doi: 10.1016/j.cognition.2018.04.002, PMID: 29653398

[ref13] Geary D. C. vanMarle K. Chu F. W. Rouder J. Hoard M. K. Nugent L. (2017). Early conceptual understanding of cardinality predicts superior school-entry number-system knowledge. Psychol. Sci. 29, 191–205. doi: 10.1177/0956797617729817, PMID: 29185879

[ref14] Göbel S. M. Watson S. E. Lervåg A. Hulme C. (2014). Children’s arithmetic development it is number knowledge, not the approximate number sense, that counts. Psychol. Sci. 25, 789–798. doi: 10.1177/0956797613516471 24482406

[ref15] Hair J. Black W. C. Babin B. J. Anderson R. E. (2010) Multivariate data analysis (7th). Upper Saddle River, New Jersey: Pearson Educational International.

[ref16] Halberda J. Feigenson L. (2008). Developmental change in the acuity of the‘number sense’: the approximate number system in 3-, 4-, 5-, and 6-year-olds andadults. Dev. Psychol. 44, 1457–1465. doi: 10.1037/a0012682, PMID: 18793076

[ref17] Halberda J. Ly R. Wilmer J. B. Naiman D. Q. Germine L. (2012). Number sense across the lifespan as revealed by a massive internet-based sample. Proc. Natl. Acad. Sci. 109, 11116–11120. doi: 10.1073/pnas.1200196109, PMID: 22733748 PMC3396479

[ref18] Halberda J. Mazzocco M. M. Feigenson L. (2008). Individual differences in non-verbal number acuity correlate with maths achievement. Nature 455, 665–668. doi: 10.1038/nature07246, PMID: 18776888

[ref19] He Y. Zhou X. Shi D. Song H. Zhang H. Shi J. (2016). New evidence on causal relationship between approximate number system (ANS) acuity and arithmetic ability in elementary-school students: a longitudinal cross-lagged analysis. Front. Psychol. 7:1052. doi: 10.3389/fpsyg.2016.01052 27462291 PMC4940382

[ref20] Holloway I. D. Ansari D. (2009). Mapping numerical magnitudes onto symbols: the numerical distance effect and individual differences in children’s mathematics achievement. J. Exp. Child Psychol. 103, 17–29. doi: 10.1016/j.jecp.2008.04.001, PMID: 18513738

[ref21] Kibbe M. M. Feigenson L. (2015). Young children ‘solve for x’using the approximate number system. Dev. Sci. 18, 38–49. doi: 10.1111/desc.12177, PMID: 24589420 PMC4394201

[ref22] Kucian K. Loenneker T. Dietrich T. Dosch M. Martin E. Von Aster M. (2006). Impaired neural networks for approximate calculation in dyscalculic children: a functional MRI study. Behav. Brain Funct. 2, 31–17. doi: 10.1186/1744-9081-2-31 16953876 PMC1574332

[ref23] Kwon N. Kim J. Kim S. Y. (2018). Effects of approximate number sense on child’s ability to solve non-symbolic multiplication problem. Korean J. Cogn. Biol. Psychol. 30, 285–291. doi: 10.22172/cogbio.2018.30.3.007

[ref001] Lee K. Park H. Lee S. (2016). A study on the structure of intelligence measured by the K-WPPSI-IV. Korean J Child Stud. 37, 107–117.

[ref24] Levy F. (1980). The development of sustained attention (vigilance) and inhibition in children: some normative data. J. Child Psychol. Psychiatry 21, 77–84. doi: 10.1111/j.1469-7610.1980.tb00018.x, PMID: 7358807

[ref25] Libertus M. E. Feigenson L. Halberda J. (2011). Preschool acuity of the approximate number system correlates with school math ability. Dev. Sci. 14, 1292–1300. doi: 10.1111/j.1467-7687.2011.01080.x, PMID: 22010889 PMC3338171

[ref26] Libertus M. E. Feigenson L. Halberda J. (2013). Is approximate number precision a stable predictor of math ability? Learn. Individ. Differ. 25, 126–133. doi: 10.1016/j.lindif.2013.02.001, PMID: 23814453 PMC3692560

[ref27] Libertus M. E. Odic D. Feigenson L. Halberda J. (2016). The precision of mapping between number words and the approximate number system predicts children’s formal math abilities. J. Exp. Child Psychol. 150, 207–226. doi: 10.1016/j.jecp.2016.06.003, PMID: 27348475 PMC4969135

[ref28] Lipton J. S. Spelke E. S. (2003). Origins of number sense: large number discrimination in human infants. Psychol. Sci. 14, 396–401. Infancy, 5, 271-290. doi: 10.1111/1467-9280.01453 12930467

[ref29] Luculano T. Tang J. Hall C. W. Butterworth B. (2008). Core information processing deficits in developmental dyscalculia and low numeracy. Dev. Sci. 11, 669–680. doi: 10.1111/j.1467-7687.2008.00716.x, PMID: 18801122

[ref30] Mazzocco M. M. Feigenson L. Halberda J. (2011). Preschoolers’ precision of the approximate number system predicts later school mathematics performance. PLoS One 6:e23749. doi: 10.1371/journal.pone.0023749, PMID: 21935362 PMC3173357

[ref31] McCrink K. Shafto P. Barth H. (2017). The relationship between non-symbolic multiplication and division in childhood. Q. J. Exp. Psychol. 70, 686–702. doi: 10.1080/17470218.2016.1151060 PMC5228418 26880261

[ref32] McCrink K. Spelke E. S. (2010). Core multiplication in childhood. Cognition 116, 204–216. doi: 10.1016/j.cognition.2010.05.003, PMID: 20537618 PMC3109428

[ref33] McCrink K. Spelke E. S. (2016). Non-symbolic division in childhood. J. Exp. Child Psychol. 142, 66–82. doi: 10.1016/j.jecp.2015.09.015, PMID: 26513326 PMC5333996

[ref34] Mussolin C. Nys J. Content A. Leybaert J. (2014). Symbolic number abilities predict later approximate number system acuity in preschool children. PLoS One 9:e91839. doi: 10.1371/journal.pone.0091839, PMID: 24637785 PMC3956743

[ref35] Obersteiner A. Reiss K. Ufer S. (2013). How training on exact or approximate mental representations of number can enhance first-grade students’ basic number processing and arithmetic skills. Learn. Instr. 23, 125–135. doi: 10.1016/j.learninstruc.2012.08.004

[ref36] Passolunghi M. C. Lanfranchi S. Altoè G. Sollazzo N. (2015). Early numerical abilities and cognitive skills in kindergarten children. J. Exp. Child Psychol. 135, 25–42. doi: 10.1016/j.jecp.2015.02.001, PMID: 25818537

[ref37] Piazza M. Facoetti A. Trussardi A. N. Berteletti I. Conte S. Lucangeli D.. (2010). Developmental trajectory of number acuity reveals a severe impairment in developmental dyscalculia. Cognition 116, 33–41. doi: 10.1016/j.cognition.2010.03.012, PMID: 20381023

[ref002] Resnick L. B. (1989). Developing mathematical knowledge. Am. Psychol. 44, 162.

[ref38] Ruff H. A. Rothbart M. K. (2001). Attention in early development: themes and variations Oxford, UK: Oxford University Press.

[ref39] Soto-Calvo E. Simmons F. R. Willis C. Adams A. M. (2015). Identifying the cognitive predictors of early counting and calculation skills: evidence from a longitudinal study. J. Exp. Child Psychol. 140, 16–37. doi: 10.1016/j.jecp.2015.06.011, PMID: 26218332

[ref40] Szkudlarek E. Brannon E. M. (2017). Does the approximate number system serve as a foundation for symbolic mathematics? Lang. Learn. Dev. 13, 171–190. doi: 10.1080/15475441.2016.1263573, PMID: 28344520 PMC5362122

[ref41] Xu F. Arriaga R. (2007). Number discrimination in 10-month-old. Br. J. Dev. Psychol. 25, 103–108. doi: 10.1348/026151005X90704

[ref42] Xu F. Spelke E. S. (2000). Large number discrimination in 6-month-old infants. Cognition 74, B1–B11. doi: 10.1016/S0010-0277(99)00066-9, PMID: 10594312

