# Corrigendum: Effects of intelligence and approximate number system on the non-symbolic division ability in preschoolers

**DOI:** 10.3389/fpsyg.2023.1330703

**Published:** 2023-11-27

**Authors:** Nayun Kwon, So-Yeon Kim

**Affiliations:** Department of Psychology, Duksung Women's University, Seoul, Republic of Korea

**Keywords:** approximate number system, mathematics, division, intelligence, cognitive development, non-symbolic division ability

In the published article, there was an error in the Funding statement. Additional funding from the National Research Foundation of Korea (NRF) was incorrectly listed. The correct Funding statement appears below.

## Funding

This work was supported by the Ministry of Education of the Republic of Korea and the National Research Foundation of Korea (NRF-2020S1A5A8044530).

The authors apologize for this error and state that this does not change the scientific conclusions of the article in any way. The original article has been updated.

